# *Acanthamoeba* and bacteria produce antimicrobials to target their counterpart

**DOI:** 10.1186/1756-3305-7-56

**Published:** 2014-01-30

**Authors:** Junaid Iqbal, Ruqaiyyah Siddiqui, Naveed Ahmed Khan

**Affiliations:** 1 Department of Biological and Biomedical Sciences, Aga Khan University, Karachi, Pakistan

## Abstract

**Background:**

In the microbial ecosystem, microbes compete for space and nutrients. Consequently, some have developed the ability to kill or inhibit the growth of other competing microbes by producing antimicrobial substances. As the ‘producer’ species are generally immune to these substances, their compounds act on the competing microbial species and give the producer more space and access to nutrients for growth. Many currently used antibiotics were developed by exploiting this potential of certain microbes.

**Findings:**

Here, the free-living amoeba, *Acanthamoeba castellanii*, was investigated for its antibacterial activity against representative Gram positive and Gram negative bacteria, while bacterial isolates were tested for their anti-amoebic properties. Conditioned medium from *A. castellanii* showed remarkable bactericidal properties against methicillin-resistant *Staphylococcus aureus* (MRSA) exhibiting almost 100% kill rate, but had limited effect against *Acinetobacter* sp., *Pseudomonas aeruginosa* and vancomycin-resistant *Enterococcus faecalis* (VRE). Similarly, the conditioned medium of *E. coli* K1 and *Enterobacter* sp., exhibited potent anti-Acanthamoebic effects in a concentration-dependent manner. Conditioned media of *Acanthamoeba*, *E. coli* K1 and *Enterobacter* sp. showed no cytotoxicity *in vitro* when tested against human brain microvascular endothelial cells. Active molecule/s in aforementioned amoebic and two bacterial conditioned media were 5 – 10 kDa, and <5 kDa respectively.

**Conclusions:**

*A. castellanii* conditioned medium showed potent bactericidal properties against MRSA. The active molecule(s) are heat- and pronase-resistant, and in the 5 to 10 kDa molecular mass range. Contrary to this, *E. coli* K1 and *Enterobacter* sp., conditioned medium showed anti-amoebic effects that are <5 kDa in molecular mass, suggestive of active metabolites.

## Findings

Infectious diseases account for 68% of global child mortality, with the vast majority occurring in developing countries [1-3]. Novel antimicrobial agents are urgently needed to counter multi-drug resistant pathogens [4]. Most clinically used antibiotics are either derived from natural products especially from microbes or semisynthetic derivatives of these molecules [5-7]. In parallel, there is a need to expand the range of organisms that can be tapped in our search to discover natural product antibiotics. Given the importance of small molecules to microbial ecology, e.g., penicillin, which is produced by *Penicillium* as a defence mechanism [8,9], it is likely that other microbes can also contribute to the discovery of new antibiotics. *Acanthamoeba* is one of the most ubiquitous protists that has been isolated from diverse environments [10]. In its natural habitat, i.e., soil and water, *Acanthamoeba* feeds on different bacteria and plays an important role in the regulation of bacterial populations [11]. However, certain bacteria especially pathogenic bacteria have developed strategies to escape the killing mechanisms of *Acanthamoeba* and instead use the amoeba as a Trojan horse or reservoir for their own benefit [12,13]. Some bacteria protect themselves from preying amoeba by secreting soluble anti-*Acanthamoeba* substances in their medium [14-16] or directly injecting such substances into *Acanthamoeba* through the type III secretory system [15]. These findings suggest that *Acanthamoeba* and Bacteria encounter each other in the environment routinely. But, how *Acanthamoeba* survive the onslaught of overwhelming bacterial population remains incompletely understood. Thus, it is reasonable to hypothesize that, in addition to bacteria, *Acanthamoeba* also possesses antibiotics to counter bacterial attack. As *Acanthamoeba* and bacteria thwart each other in their natural habitat, the overall aim of this study was to determine the anti-bacterial activities of *Acanthamoeba* and also the anti-Acanthamoebic properties of selected bacterial strains.

A keratitis isolate of *Acanthamoeba castellanii* was obtained from the American Type Culture Collection (50492) and grown axenically in PYG medium [proteose peptone 0.75% (w/v), yeast extracts 0.75% (w/v) and glucose 1.5% (w/v)] in tissue culture flasks aerobically at 30°C without shaking [17-19]. *A. castellanii* conditioned media (ACM) were prepared by incubating confluent cultures (~2×10^6^ trophozoites/mL) for 48 h in PYG. Given confluent cultures, amoebae number increased slightly to ~3×10^6^ trophozoites/mL. Next, cell-free medium was collected by centrifugation (1000 × g for 5 min) and filtered using 0.22 μM pore size filters. In some experiments, the ACM was treated at 95°C for 10 min to inactivate *A. castellanii* extracellular proteases.

Bacterial isolates used in the present study were *Acinetobacter* sp., *Aeromonas hydrophila, Enterobacter aerogenes, Enterobacter* sp., *Escherichia coli* K1, *Klebsiella pneumoniae,* Methicillin-resistant *Staphylococcus aureus* (MRSA), *Pseudomonas aeruginosa*, *Shigella flexneri,* and Vancomycin-resistant *Enterococcus faecalis* (VRE). *Escherichia coli* K1 strain RS218 (O18:K1:H7) and MRSA have been described previously [20,21]. They were isolated from the cerebrospinal fluid of a meningitis patient [20] and blood culture of septicemia patient [21], respectively. *Enterobacter* sp. was isolated as a contaminant in *Acanthamoeba* culture, and all other bacteria are clinical isolates obtained from the Aga Khan University Hospital, Karachi (available upon request). Bacterial conditioned media (BCM) were prepared by culturing single colonies (grown on nutrient agar plates) in 100 mL PYG medium and incubating for 24 h at 37°C aerobically, with shaking. These conditions resulted in growth of all bacterial cultures to stationary phase of their growth. The bacterial cultures were centrifuged at 4000 × g at 4°C for 20 min. The supernatants were collected and filter sterilized using 0.22 μM pore size membrane filters. In some experiments, BCM were treated at 95°C for 10 min to inactivate extracellular enzymes.

### Bacterial conditioned media inhibited *A. castellanii* growth

**Figure 1:**
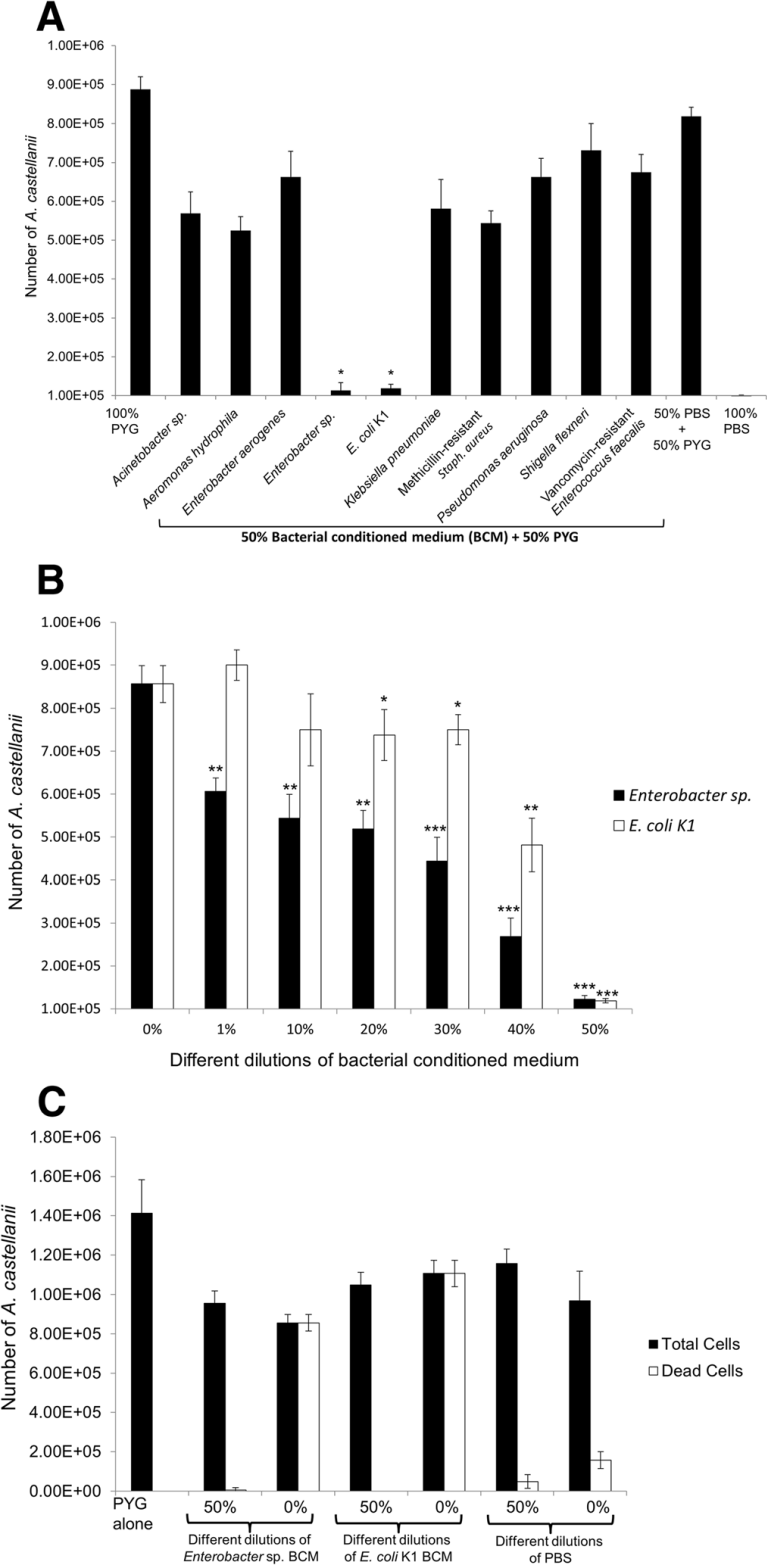
**The conditioned media of *Enterobacter *sp. and *Escherichia coli *K1 exhibited amoebistatic and amoebicidal activities. (A)***A. castellanii* (10^5^) were incubated with 50% bacterial conditioned media (BCM) and 50% PYG at 30°C for 48 h. For controls, *A. castellanii* were incubated with PYG alone, 100% PBS, and 50% PBS plus 50% PYG. After incubation, amoebae were counted using a haemocytometer. The initial inoculum of 10^5^ amoebae was used as baseline. Note that the incubation in the non-nutritive 100% PBS showed no growth, while incubation in the growth medium resulted in over 8-fold increase in the number of amoebae compared with the original inoculum. P-values were calculated by comparing results of 100% PYG with BCM using student’s t-test. (*) indicates p<0.001. **(B)***Enterobacter* sp. and *E. coli* K1 BCM were diluted using PYG, where 0% dilution represents neat BCM, while 50% dilution represents BCM and PYG in 1:1 ratio. *A. castellanii* (10^5^) were incubated in various dilutions of BCM of *Enterobacter* sp. and *E. coli* K1 and incubated at 30°C for 48 h. After incubation, amoebae were counted using a haemocytometer. The initial inoculum of 10^5^ amoebae increased to over 8-fold in PYG alone (0% dilution). P-values were calculated by comparing results of 0% BCM (i.e., PYG alone) with different concentrations of BCM. (*), (**) and (***) indicate *p-*values of <0.05, <0.01 and <0.001, respectively. **(C)***A. castellanii* (10^6^) were incubated with neat (0%) or diluted (50%) BCM at 30°C for 24 h. After incubation, 0.1% Trypan blue dye was added and amoebae along with the blue coloured amoebae (dead cells) were counted using a haemocytometer. Note that cells treated with neat BCM produced *A. castellanii* death. The data are presented as the mean ± standard error of three independent experiments performed in duplicate.

To determine amoebistatic activity of BCM, *A. castellanii* trophozoites (10^5^ amoebae/0.5 mL/well) were incubated in PYG with different amounts of BCM in 24 well plates at 30°C for 48 h. After this incubation, amoebae were counted using a haemocytometer. In controls, *A. castellanii* trophozoites were incubated in PBS (non-nutrient) and exhibited no growth (Figure 1A), while *A. castellanii* incubated in PYG (growth medium) exhibited a nine-fold increase in amoebae numbers from the original inoculum (Figure 1A). Among the different bacterial isolates tested, *Enterobacter* sp., and *E. coli* K1 BCM inhibited *Acanthamoeba* growth (ca. 98% and 97% amoebistatic effects respectively). To determine whether amoebistatic effects of BCM were due to nutrient-depletion, *A. castellanii* trophozoites (10^5^ amoebae in 250 μL PYG) were incubated with 250 μL of PBS in a 1:1 ratio. The results revealed amoebae growth profiles, in 50% PBS, similar to amoebae incubated in neat PYG (Figure 1A). Among other bacteria, BCM of *Acinetobacter* sp., *A. hydrophila*, *K. pneumoniae* and MRSA also inhibited *A. castellanii* growth (ca. 40%, 35%, 33% and 38% amoebistatic effects respectively). The conditioned media showed amoebistatic properties, indicating the water soluble nature of the bioactive molecule(s). In order to address the possibility of conversion of *Acanthamoeba* trophozoites into cysts after BCM treatment, BCM-treated *Acanthamoeba* trophozoites were incubated with 0.5% SDS for 10 min, which is known to selectively lyse trophozoites [22]. Post-SDS treatment, none of the BCM-treated *Acanthamoeba* exhibited cyst presence. To determine the potency of the compound(s) in the conditioned media of *Enterobacter* sp., and *E. coli* K1, serial dilutions of the BCM were made and tested against *A. castellanii*. The results demonstrated that BCM of *Enterobacter* sp. exhibited amoebistatic effects in a concentration-dependent manner (Figure 1B). At 50% dilution, *Enterobacter* sp. BCM exhibited potent amoebistatic effects (ca. 97%) (Figure 1B), and similarly *E. coli* K1 BCM exhibited more than 95% amoebistatic effects (Figure 1B). However, *Enterobacter* sp. BCM was found to be more potent in inhibiting *A. castellanii* even at lower dilutions, compared with *E. coli* K1 BCM (Figure 1B).

### *Enterobacter* sp. and *E. coli* K1 conditioned media produced *A. castellanii* death

For amoebicidal activity of BCM, *A. castellanii* trophozoites (10^6^ amoebae/0.5 mL/well) were incubated in PYG with different amounts of BCM in 24 well plates at 30°C for 48 h. Following this incubation, amoebae viability was determined by adding 0.1% Trypan blue and number of live (non-stained) and dead (stained) *A. castellanii* were enumerated using a hemocytometer. At 50% concentration, *E. coli* K1 and *Enterobacter* sp. BCM did not affect *A. castellanii* viability (0% and 0.7% respectively), while at 100% concentration, BCM of both *E. coli* K1 and *Enterobacter* sp. exhibited more than 90% amoebicidal effects (Figure 1C).

### Anti-Acanthamoebic compound(s) in *Enterobacter* sp. and *E. coli* K1 conditioned media are pronase-resistant and <5 kDa in molecular mass

**Figure 2:**
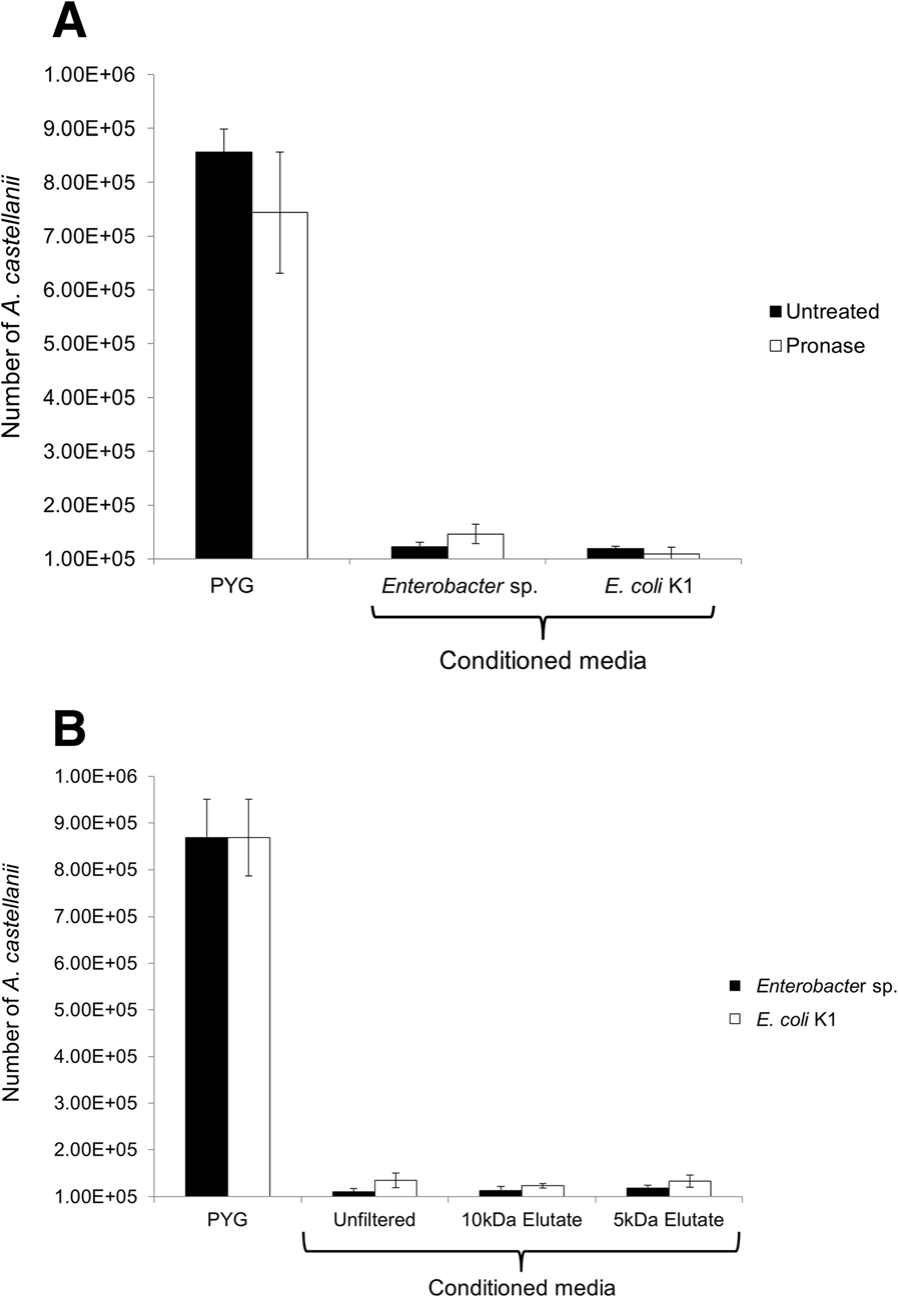
**Anti-Acanthamoebic activities of conditioned media of *Enterobacter *sp. and *E. coli *K1 are resistant to pronase digestion and smaller than 5 kDa in molecular mass. (A)** The conditioned media of *Enterobacter* sp. and *E. coli* K1 were prepared in PYG. The conditioned media were diluted in PYG in a 1:1 ratio and treated with 1 mg per mL of Pronase at 37°C for 4 h. Next, *A. castellanii* cells (10^5^) were incubated with Pronase-treated and untreated conditioned media at 30°C for 48 h. After incubation, *A. castellanii* were counted using a haemocytometer. The initial inoculum of 10^5^*A. castellanii* was used as a baseline, which increased to over 8-fold in PYG alone. Note that Pronase-treatment had no effect on growth inhibitory effects of conditioned media. **(B)***Enterobacter* sp. and *E. coli* K1 conditioned media were filtered through 10 kDa and 5 kDa MWCO Spin-X UF. The eluate and neat conditioned media (unfiltered) were incubated with *A. castellanii* cells (10^5^) for 48 h at 30°C. After incubation, *A. castellanii* were counted using a haemocytometer. The initial inoculum of 10^5^*A. castellanii* was used as a baseline, which increased to over 8-fold in PYG alone. Note that eluate from 5 kDa filters exhibited growth inhibitory effects at levels similar to neat conditioned media. The data are presented as the mean ± standard error of three independent experiments performed in duplicate.

To determine the molecular mass of the active molecule(s), conditioned media of *Enterobacter* sp. and *E. coli* K1 were filtered through 10 kDa and 5 kDa molecular weight cut off Spin-X UF columns (Corning). Both the eluate and retentate were used in the aforementioned antimicrobial assays. To determine the chemical nature of active substance(s), conditioned media were treated with 1 mg per mL pronase, mixture of broad spectrum proteases, at 37°C for 4 h. Antimicrobial activity of pronase-treated conditioned media were determined as described above. Treatment of pronase did not block *A. castellanii* growth inhibitory activities of BCM (Figure 2A). To determine the approximate mass of active molecules, BCM were filtered through 10 kDa and 5 kDa size exclusion spin columns. The results revealed that eluate of both 5 kDa and 10 kDa exhibited *A. castellanii* growth suppression activities, suggesting that the active molecule(s) may be secondary metabolite(s) (Figure 2B).

### *Enterobacter* sp. and *E. coli* K1 conditioned media are not cytotoxic to primary human brain microvascular endothelial cells

Cytotoxicity assays were performed as previously described [20]. Briefly, assays were performed in 24 well plates containing confluent human brain microvascular endothelial cells (HBMEC) monolayers. The conditioned media (250 μL) or PYG medium (250 μL) were added in 250 μL RPMI-1640. Plates were incubated at 37°C in a 5% CO_2_ incubator and monitored for monolayer disruptions over the period of 24 h. After this incubation, the supernatants were collected from each well, centrifuged to remove cellular debris and then cytotoxic effects were determined by estimating the amount of lactate dehydrogenase release from HBMEC using a Cytotoxicity Detection kit (Roche Applied Sciences). The percent cytotoxicity was calculated as follows: % cytotoxicity = (sample value – control value) / (total LDH release – control value) × 100. Control values were determined by incubating HBMEC monolayers with RPMI 1640 alone and total LDH release was obtained by completely lysing the HBMEC using 1% Triton X-100. Cytotoxicity assays were performed to determine the effects of the *Enterobacter* sp., and *E. coli* K1 BCM on primary HBMEC. Neither *E. coli* K1 BMC nor *Enterobacter* sp. BCM produced host cell cytotoxicity (0.3% and 2% host cell death respectively). As BCM was prepared using PYG, similar volume of PYG alone (250 μL) were used as controls. The results revealed that PYG alone exhibited neither monolayer disruptions nor HBMEC cytotoxicity (ca. 2%).

### *A. castellanii* conditioned media exhibited potent antibacterial activities against MRSA

To determine the effects of ACM on multiple drug resistant (MDR) bacteria, clinical isolates of *Acinetobacter* sp., *P. aeruginosa*, MRSA and VRE were used. For antibacterial screening, approximately 10^6^ colony forming units (c.f.u), suspended in 10 μLwere incubated with 190 μL of ACM at 37°C for 18 h. After incubation, cultures were ten-fold serially diluted in PBS and plated on agar plates. Plates were incubated at 37°C, overnight and bacterial c.f.u. were enumerated [7]. For controls, bacteria were incubated in PYG alone and PBS alone. The results revealed that ACM killed more than 99% MRSA and exhibited 8% bactericidal effects against VRE, but had no effect against other bacteria tested (Table 1). Heating at 95°C for 10 min had no effect on the bactericidal properties of ACM. All subsequent experiments were performed using heated-ACM against MRSA. Serial dilutions showed that aliquots containing 190 μL ACM exhibited 100% bactericidal effects, 180 μL ACM showed 93.3% bactericidal effects, whereas dilution of 70%: 30% (ACM:PYG) had no bactericidal activity (Table 1). Antibacterial activity was observed in the eluate after passing ACM through 10 kDa but retained by 5 kDA size-exclusion spin columns, indicating that active ingredient(s) are between 5 to 10 kDa in molecular mass. The treatment with pronase had no effect on bactericidal activity of ACM (Table 1). Notably, bacterial growth in PYG medium alone is comparable to LB (data not shown). The ACM had no cytotoxic effects on HBMECs in cytotoxicity assays (data not shown).

**Table 1:** ***Acanthamoeba *** **conditioned medium (ACM) exhibited selective antibacterial properties**

**Bacteria +** ***Acanthamoeba *** **conditioned medium (ACM)**	**CFU remaining**^**a **^**(Bacteria + ACM)**	**Bacterial kill (%)**
Methicillin-resistant *Staphylococcus aureus* (MRSA, 10 μL) + ACM (190 μL)	0	100
*Acinetobacter* (10 μL) + ACM (190 μL)	1 × 10^10^	0
*Pseudomonas aeruginosa* (10 μL) + ACM (190 μL)	6 × 10^9^	0
Vancomycin resistant *Enterococcus faecalis* (VRE, 10 μL) + ACM (190 μL)	3.75 × 10^5^	8.12 ± 9.8
MRSA (10 μL) + heated ACM (190 μL)	0	100
MRSA (10 μL) + heated ACM (180 μL) + PYG (10 μL)	6.65 × 10^4^	93.3 ± 9.9
MRSA (10 μL) + heated ACM (140 μL) + PYG (50 μL)	1.55 × 10^7^	0
MRSA (10 μL) + heated ACM eluate of 10 kDa spin exclusion column (190 μL)	0	100
MRSA (10 μL) + heated ACM eluatee of 5 kDa spin exclusion column (190 μL)	1.34 × 10^10^	0
MRSA (10 μL) + heated-pronase-treated ACM (190 μL)	0	100

The data are presented as the mean ± SE of three independent experiments. ^a^An inoculum of ~10^6^ bacterial c.f.u. was used.

The presence of antimicrobials in microbes is not a novel concept and they are known to be an ancient weapon in the defense against the growth of other microbes. Since the discovery of penicillin produced in nature by mould, *Penicillium* in 1928 [8], a large number of microbes have been tested for the isolation of potentially useful antimicrobials, while there has been renewed interest in finding new antibiotics from unique sources such as actinomycetes, cyanobacteria and unculturable bacteria, in addition to known sources such as streptomycetes.

The ability of bacteria to thrive at high population densities and the predatory role of *Acanthamoeba* in the control of bacterial populations in the environment as well as the ability of the amoeba to act as a “Trojan horse” of the microbial world suggests that bacteria-protists are involved in convoluted interactions [11-16]. The precise nature of such complex interactions is not clear, but it is shown here that both bacteria and amoebae are able to counter-attack each other and the overwhelming microbial densities by secreting/harboring antimicrobials. Among four bacteria tested, it is interesting that ACM exhibited selective and potent activity against MRSA. Superbugs, such as MRSA have shown the ability to cause untreatable infections and have become a major threat in our fight against bacterial infections [23]. The selective and potent nature of ACM against Gram-positive MRSA and partly against VRE, but not against Gram-negative *P. aeruginosa* is interesting. It is possible that *P. aeruginosa* and *Acanthamoeba* are involved in complex interactions where the bacteria can employ strategies to defend itself and counter amoebic attack [23]. Future studies will test ACM against a large panel of diverse bacterial pathogens to determine broad-spectrum or species-specific antibacterial properties. The discovery of antibacterial compound(s) from *Acanthamoeba* will open avenues for future studies in other free living amoebae/protists as a potential source of antibiotics for the treatment of emerging bacterial pathogens such as MRSA. Because of increasing trends in antibiotic resistance in different pathogenic bacteria [2], the present findings are promising in our efforts to treat infections caused by drug-resistant bacteria.

Similarly, among various bacterial isolates tested, BCM of *Enterobacter* sp. and *E. coli* K1 exhibited measurable amoebistatic and amoebicidal properties. Future studies will address the question of whether these BCM have selective action against *Acanthamoeba* or have wide scale activities against different protist pathogens. Interestingly, neither ACM nor BCM were cytotoxic to brain endothelial cells in vitro, suggestive of the selective action of these conditioned media against MRSA and *Acanthamoeba*, respectively. Future studies will further test the specificity and the absence of putative target(s) in human cells using primary cells of various origins. Overall, the present findings are promising in our search to find additional sources of antimicrobials with novel modes of action to confront this menace. Work is currently underway to characterize further the antimicrobial properties of ACM and BCM and active components, possibly novel secondary metabolites in the case of BCM, being resistant to pronase and heat treatment and less than 5 kDa in molecular mass. As antimicrobial resistance is on the increase, finding novel non-toxic antimicrobial compound(s) from a variety of species prevalent in clinical settings is crucial. It is hoped that these molecules will eventually be developed into treatments for bacterial and amoebic infections that are increasingly resistant to currently available drugs, however, this will require intensive research in the next few years.

## Competing interests

The authors declare that they have no competing interests.

## Authors’ contributions

NK conceived the study. JI and RS designed and conducted all experiments under the supervision of NAK. RS, JI and NAK contributed to the writing of the manuscript. All authors approved the final manuscript.
